# Condiciones de salud de los indígenas hitnü potencialmente expuestos a petróleo crudo (Arauca, Colombia)

**DOI:** 10.7705/biomedica.6591

**Published:** 2022-12-01

**Authors:** Carolina Rivero-Rubio, Angélica I. Navarro-Rodríguez, María C. Castro-Reyes, Óscar Araújo-Quintana, José Moreno-Montoya, Jesús A. Estévez-García, Pablo A. Martínez-Silva, Álvaro J. Idrovo, Claudia Amaya-Castellanos

**Affiliations:** 1 Departamento de Salud Pública, Escuela de Medicina, Universidad Industrial de Santander, Bucaramanga, Colombia Universidad Industrial de Santander Departamento de Salud Pública Escuela de Medicina Universidad Industrial de Santander Bucaramanga Colombia; 2 Subdirección de Estudios Clínicos, Fundación Santa Fe de Bogotá, Bogotá, D.C., Colombia Universidad Nacional de Colombia Subdirección de Estudios Clínicos Fundación Santa Fe de Bogotá Bogotá, D.C. Colombia; 3 Centro de Investigación en Salud Poblacional, Instituto Nacional de Salud Pública, Cuernavaca, México Instituto Nacional de Salud Pública Centro de Investigación en Salud Poblacional Instituto Nacional de Salud Pública Cuernavaca Mexico; 4 Sinergias - Alianzas Estratégicas para la Salud y el Desarrollo Social, Bogotá, D.C., Colombia Sinergias - Alianzas Estratégicas para la Salud y el Desarrollo Social Bogotá, D.C. Colombia

**Keywords:** salud de poblaciones indígenas, contaminación por petróleo, salud ambiental, perfil de salud, cáncer, epidemiología, Health of indigenous peoples, petroleum pollution, environmental health, health profile, cancer, epidemiology

## Abstract

**Introducción.:**

El pueblo hitnü vive en condiciones sanitarias precarias, con inseguridad alimentaria y víctima de la violencia sociopolítica en Arauca (Colombia). Además, se sospecha que pueden estar afectados por la exposición a los hidrocarburos del petróleo.

**Objetivo.:**

Identificar los eventos de salud del perfil de morbilidad y mortalidad de los indígenas hitnü que podrían asociarse con la exposición a petróleo crudo.

**Materiales y métodos.:**

Se realizó un estudio transversal con indígenas hitnü, durante febrero y marzo de 2021, época de sequía. Se aplicó un cuestionario de hogares y uno individual para recolectar datos del ambiente peridomiciliario, ocupaciones y otras actividades, así como datos sociodemográficos, signos, síntomas y hallazgos de un examen médico.

La potencial asociación con los hidrocarburos se exploró considerando tres grupos, según su localización: cabecera de Arauca, resguardo Aspejená (no expuestos) y resguardos de San José del Lipa y La Vorágine (expuestos por su cercanía al río Ele y afluentes). Con listados libres, se exploraron las causas de muerte. El estudio incorporó un riguroso manejo intercultural en todos sus componentes.

**Resultados.:**

Participaron 576 indígenas de 16 asentamientos. El agua consumida pudo servir como medio de exposición a los hidrocarburos. Los problemas de salud fueron muy variados e incluían enfermedades infecciosas y crónicas, malnutrición y trauma. Las masas en el cuello se asociaron con residir en los resguardos ancestrales (RP=3,86; IC_95%_ 1,77-8,39), territorios potencialmente expuestos al petróleo. Las causas de muerte más relevantes fueron el homicidio, los tumores y la tuberculosis.

**Conclusión:**

Por su posible asociación con los hidrocarburos, es prioritario el estudio intercultural de linfoadenopatías entre indígenas potencialmente expuestos al petróleo.

## Abstract (en)

**Introduction:**

The Hitnü indigenous people live in precarious sanitary conditions, with food insecurity and being victims of sociopolitical violence in Arauca, Colombia. In addition, it is possible that they may be affected by exposure to hydrocarbons found in oil.

**Objective:**

To identify the health outcomes of morbidity and mortality profiles of the Hitnü people that could be associated with the exposure to crude oil.

**Materials and methods:**

A cross-sectional study was carried out with Hitnü indigenous people, during February and March, 2021, time of drought. A household questionnaire was applied, and one individual to collect data from the environment around the house, occupations and other activities, as well as data from sociodemographic, signs, symptoms, and findings of a medical examination. The potential association with hydrocarbons was explored considering three groups: inhabitants in Arauca city, Aspejaná reserve (not exposed), and San José del Lipa and La Vorágine reserves (exposed by the Ele river and tributaries). With free listings, causes of death were explored. The study incorporated a rigorous intercultural management in all its components.

**Results:**

A total of 576 indigenous people from 16 settlements participated. The water consumed could serve as means of exposure to hydrocarbons. Health problems were very varied, including infectious and chronic diseases, malnutrition, and trauma. The masses on the neck were associated with residing in the ancestral reserves (PR = 3.86; CI95% 1.77-8.39), territories with potential exposure to crude-oil. The most relevant causes of death were homicide, tumors, and tuberculosis.

**Conclusion:**

For its possible association with exposure to hydrocarbons, it is a priority to start the intercultural study of lymphadenopathies in indigenous communities potentially exposed to crude oil.

El petróleo crudo está constituido por una mezcla de sustancias químicas, entre las que sobresalen por su toxicidad el benceno, el xileno, el tolueno y otros hidrocarburos aromáticos policíclicos ^1^. Debido a estos compuestos y sus mezclas, la extracción petrolera se ha asociado con el incremento de diversos efectos adversos para la salud, entre los que sobresalen los daños hematológicos, los cromosómicos y otras expresiones de genotoxicidad, abortos espontáneos, deterioro de la calidad del semen y trastornos respiratorios diversos, así como cambios conductuales, específicamente entre menores de edad residentes en lugares con derrames de petróleo ^2^.

Reconociendo que pueden existir diferencias con los grupos indígenas amazónicos, en una revisión reciente se sugirió que las actividades de extracción petrolera en estas comunidades se asocian con neoplasias, especialmente, cáncer en estómago, recto, piel, tejidos blandos, riñones y cuello uterino en adultos, y leucemia entre menores de edad ^3^.

En Colombia, la extracción petrolera es un importante generador de divisas, siendo el departamento de Arauca una de las zonas con mayor producción, pese al contexto de conflicto armado existente desde décadas atrás ^4^. Tres de los campos petroleros más importantes de esta región son: Caño Limón, Caricare y Chipirón; este último se encuentra en medio de la estera de Lipa y ha sido el detonante de quejas de diversos líderes sociales y de un conflicto socioambiental aún vigente. Véase: https://ejatlas.org/conflict/destruccion-ecosistema-bioestrategico-el-lipa-arauca-colombia

Los esteros son ambientes acuáticos de zonas planas y bajas que, debido a sus múltiples conexiones con caños, se inundan en épocas lluviosas. Dado el ecosistema de esteras, en el caso del campo petrolero Caricare, existe la posibilidad de que las piscinas presentes en los campos petroleros viertan su contenido en los esteros, especialmente en épocas lluviosas, y que de allí pase al río Ele (figura 1).

**Figura 1:**
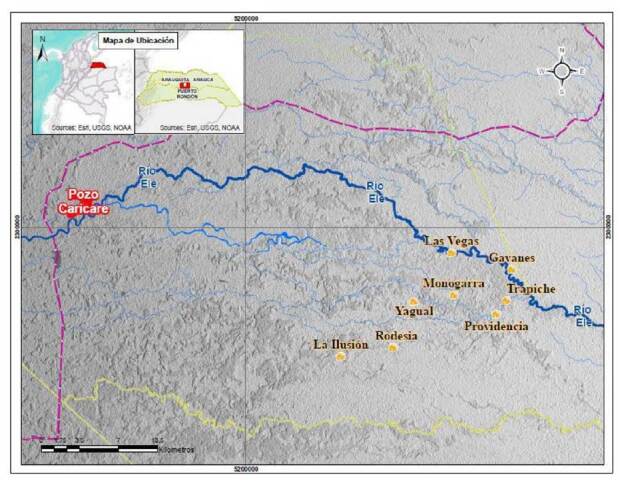
Ubicación de las comunidades hitnü en los territorios ancestrales y su relación con el pozo Caricare. El río Ele y sus afluentes en una zona de esteros de la Orinoquia colombiana, durante periodos de sequía

Fuente: Grupo de Geomática, Universidad Industrial de Santander

Debido a las condiciones precarias de los indígenas hitnü identificadas por funcionarios públicos que estuvieron años atrás en sus territorios, en 2017, un juez solicitó al Ministerio de Salud y Protección Social adelantar un estudio para valorar más detalladamente la situación ^5^, labor que fue delegada a instituciones académicas y de salud. El diseño original del estudio fue hecho por dependencias del Ministerio de Salud y Protección Social, con un enfoque totalmente epidemiológico, por lo que el equipo investigador incorporó otros componentes metodológicos, con el fin de hacerlo más sólido en la medición de la exposición a hidrocarburos y la búsqueda de efectos adversos.

El presente estudio epidemiológico hace parte de este proyecto de salud ambiental, y corresponde a la aproximación panorámica de las condiciones de salud del pueblo hitnü, que buscó identificar y seleccionar los eventos de salud que podrían ser explorados de manera más detallada. Se espera que el enfoque de identificación de posibles efectos adversos de salud ambiental usado en esta experiencia sirva de ejemplo a futuros estudios similares en poblaciones en situación de vulnerabilidad y de difícil acceso.

## Materiales y métodos

### Contexto

El pueblo hitnü es un grupo indígena de tradición nómada que, debido a las actividades de ganadería y agricultura de los colonos, la extracción petrolera y los grupos armados ilegales, se ha asentado en territorios reducidos y en condiciones muy precarias ^6^. Los territorios ancestrales, todavía ocupados por las comunidades hitnü, están ubicados en los resguardos de San José del Lipa y La Vorágine, en los municipios de Arauca y Arauquita, ambos en el departamento de Arauca ^7^^,^^8^. Además, hay un grupo creciente de indígenas asentado en la cabecera municipal de Arauca, en condiciones muy precarias, y otro grupo, en el recién creado resguardo de Aspejená en el municipio de Puerto Rondón, donde se han ubicado con el propósito de escapar de diversos hechos de violencia sociopolítica.

Actualmente, las comunidades hitnü tienen relación estrecha con el río Ele, y otras fuentes de agua relacionadas, por lo que es posible que la contaminación generada en el campo petrolero Caricare llegue por medio del agua a los individuos residentes en sus riberas, aproximadamente 30 km río abajo. Otras formas de exposición, como la vía aérea, no se consideraron relevantes en este caso debido a los hallazgos de un estudio ambiental previo (Amaya-Castellanos CI, Idrovo AJ. Petróleo, despojo y enfermedad entre el pueblo Hitnü. Una experiencia de investigación intercultural en salud ambiental. Bucaramanga: Universidad Industrial de Santander). Además, se han registrado y observado cambios en la coloración del río, junto con manchas oleosas que fueron reportadas durante la exploración etnográfica de este estudio. Esto puede ser similar a lo ocurrido en el río Arauca y sus afluentes, por donde se transportaron hidrocarburos muchos kilómetros río abajo de donde ocurrieron derrames o explosiones de oleoductos ^9^.

Además, es importante señalar que el cambio cultural impuesto al pueblo hitnü, al pasar del nomadismo al sedentarismo de manera rápida, ha tenido un enorme impacto en sus formas de vida. Esto ha generado serios problemas de saneamiento básico e inseguridad alimentaria, junto a problemas de violencia intracomunitaria, con los colonos y con grupos al margen de la ley, así como el incremento en la prevalencia de enfermedades infecciosas. Esta situación es similar a la ocurrida con el pueblo nukak en el departamento del Guaviare ^10^, y a la que sufrió el pueblo u’wa en el nororiente colombiano ^11^.

### Diseño, población y muestra

Se realizó un estudio de corte transversal con indígenas de distintas edades que aceptaron participar voluntariamente, constituyendo así, una muestra significativa de la población hitnü. Si bien se tenían datos del último censo nacional de 2019 (n=513), con el fin de realizar un muestreo representativo, después de contactar a algunos representantes indígenas se identificó que era culturalmente más adecuado hacer un censo *in situ* como parte del estudio (n=658, durante el trabajo de campo). Esta sería una oportunidad única de conocer su condición de salud, algo útil para la comunidad, debido a las grandes dificultades de acceso a los servicios de salud. De esta manera, durante el trabajo de campo se buscó actualizar los datos poblacionales y de salud, quedando por fuera del censo únicamente quienes no se encontraban en los asentamientos en el momento del trabajo de campo o aquellos que libremente decidían no participar.

### Recolección de datos

Por causa de la pandemia de COVID-19, el trabajo de campo tuvo que esperar a que los gobiernos nacional y departamental, así como las autoridades indígenas, permitieran el ingreso al territorio. De esta manera, el trabajo de campo se llevó a cabo entre mediados de febrero y comienzos de marzo de 2021, época que corresponde a la temporada sin lluvias. Esto facilitó el acceso por vía terrestre y tuvo como consecuencia que los problemas de salud asociados a las temporadas lluviosas fueran menos frecuentes, incluyendo los de aparición aguda, posiblemente relacionados con la exposición hidrocarburos por vía hídrica.

Los datos fueron recolectados en tres tipos de instrumentos: uno para datos de vivienda y familia, que fue contestado por el jefe del hogar; el segundo, para recolectar datos individuales, contestado por cada participante o el encargado de su cuidado, y el tercero fue una guía de examen físico, que fue diligenciado por un profesional de la medicina.

En el cuestionario sobre vivienda y familia, se indagó sobre ubicación de la vivienda, número de integrantes de la familia, materiales de construcción de la vivienda, servicios disponibles, características de la cocina, acceso y tipo de agua para consumo, actividades para obtener el alimento familiar y casos de familiares fallecidos en los últimos diez años.

El instrumento individual tuvo un componente general, y unos ítems especiales para los menores de edad y las mujeres. En este, se indagó el sexo, la edad, la calidad de la convivencia, la dependencia económica y social, el alfabetismo, las ocupaciones, y el consumo de alcohol, cigarrillo, café y yopo (sustancia alucinógena de origen vegetal, usada en rituales). De igual manera, se obtuvo información sobre la condición de salud general, signos y síntomas variados en los diferentes sistemas y órganos corporales. En el caso de las mujeres, se adicionaron ítems que permitieran obtener información sobre la menarquia, la menstruación, los embarazos previos y resultados reproductivos.

El examen físico incluyó signos vitales, un examen por segmentos corporales (cabeza, tronco y miembros) y por sistemas; se incluyó un apartado amplio para los resultados de la búsqueda de ganglios linfáticos, y un diagnóstico presuntivo, cuando los médicos así lo consideraron.

El instrumento usado para recolectar datos se elaboró en español, porque no existe escritura en el pueblo hitnü ^12^. Para un mejor entendimiento de las preguntas, los encuestadores leían las preguntas en español y las explicaban si era requerido, incluso usando expresiones faciales o señales con las manos. La comprensión de los cuestionarios sobre el hogar y el individual, fueron validados con indígenas.

Quienes recolectaron la información mediante cuestionario fueron dos indígenas con estudios universitarios (una sikuani y otro inga), y una auxiliar de enfermería. Las tres personas eran conocidas por la comunidad hitnü e, incluso, fueron recomendados por ellos mismos por la confianza que les generaban y la mayor facilidad para la comunicación oral.

Los exámenes físicos fueron practicados por médicos graduados, dos mujeres y un hombre, previamente entrenados para hacerlo de manera estandarizada, siguiendo un instrumento diseñado *ad hoc* por un médico toxicólogo experto en salud ambiental. En todo momento, el equipo investigador estuvo acompañado por un médico con reconocida experiencia con indígenas a nivel nacional, una antropóloga y una trabajadora social; entre sus actividades estuvo el asesorar y cuidar para que hubiese respeto hacia la cultura hitnü en todos los momentos del estudio, pero, con especial énfasis, durante el trabajo de campo por el contacto estrecho. Además, los líderes del equipo investigador ya contaban con experiencia previa en estudios con población indígena.

Siguiendo las costumbres del pueblo hitnü, la convocatoria a participar en el estudio estuvo acompañada de una “olla comunitaria”, en la que el equipo investigador llevaba la comida (con preferencia por la carne de res, a la que suelen tener poco acceso), o se la compraba a los mismos indígenas. Además, durante el trabajo de campo, se requirió la participación de traductores que acompañaron a los investigadores en sus diversas actividades, así como, el apoyo de lancheros para la movilidad fluvial (ríos y caños), y el transporte hasta algunos asentamientos distantes. Las cocineras y leñadores encargados de cocinar los alimentos de la “olla comunitaria”, fueron indígenas hitnü que recibieron un pago económico por sus servicios.

Cuando se requirió, se hicieron trueques de participación con objetos considerados útiles entre indígenas e investigadores. Durante el trabajo de campo, los investigadores vivieron en similares condiciones a las de los indígenas, incluso, durmieron en viviendas sin muros (características de los indígenas hitnü) o en casas de colonos. Mayores detalles sobre este tema se encuentran en otro escrito (Amaya-Castellanos CI, Idrovo AJ. Petróleo, despojo y enfermedad entre el pueblo Hitnü. Una experiencia de investigación intercultural en salud ambiental. Bucaramanga: Universidad Industrial de Santander).

### Medición de la exposición

Dado el carácter exploratorio del presente estudio sobre la exposición al petróleo crudo o a las actividades relacionadas con el proceso extractivo, se optó por una aproximación en los asentamientos, que corresponden a la región donde suelen realizar actividades cotidianas, incluyendo las que implican contacto con el agua; de esta manera, los asentamientos fueron clasificados en tres grupos, así:

- los localizados en los resguardos de San José de Lipa y La Vorágine, en los municipios de Arauca y Arauquita, territorios ancestrales del pueblo hitnü. Las condiciones de saneamiento básico y seguridad alimentaria eran variadas, pero, dado que por allí pasa el río Ele, es posible que se hubiera presentado exposición al petróleo crudo u otros químicos usados en su extracción.
- los localizados en la cabecera municipal de Arauca, que incluyó a individuos que habían sido desplazados violentamente de sus territorios (asesinato de sus líderes) y tenían problemas de saneamiento básico e inseguridad alimentaria de diversa magnitud. Se supone que no habían estado expuestos recientemente a las mezclas de petróleo crudo o a las sustancias usadas en su extracción.
- los localizados en el resguardo de Aspejená, en el municipio de Puerto Rondón, donde se encuentran individuos que fueron desplazados violentamente de sus territorios originales, y que han sido reubicados lejos de posibles fuentes de exposición a mezclas de petróleo crudo o sustancias usadas en su extracción. Sus condiciones de saneamiento básico y alimentación son mejores que en los asentamientos anteriores, y están alejados de la cabecera municipal.

### Resultados y covariables

Partiendo del supuesto de que en salud ambiental suele ser difícil encontrar asociaciones entre exposición y eventos en salud, por la poca frecuencia de estos últimos y las dificultades de medir apropiadamente la exposición ^13^^,^^14^, los instrumentos del estudio se diseñaron, específicamente para explorar e identificar varios eventos de salud, con potencial de ser estudiados con el tamaño de muestra disponible en el contexto de un estudio transversal. Si bien se exploraron muchos síntomas y eventos autorreportados, así como signos identificados por los médicos, por motivos de espacio, únicamente se presenta un número limitado de datos que sirvan para vislumbrar el perfil general de morbilidad del pueblo hitnü, y para mostrar la lógica utilizada para seleccionar los eventos adversos que requieren un análisis específico para determinar su potencial asociación con los hidrocarburos del petróleo provenientes del pozo Caricare.

### Métodos estadísticos y listados libres

Inicialmente, se describieron las variables categóricas mediante porcentajes, y las cuantitativas con medidas de tendencia central y dispersión, según la distribución observada. Esto permitió hacer comparaciones de ocurrencia entre los grupos de interés, e incorporar los hallazgos en el algoritmo de identificación de eventos adversos potencialmente asociados con la exposición a hidrocarburos en la explotación petrolera del pozo Caricare.

Es importante señalar que hubo variables que no fueron respondidas por todos los participantes, ocasionando la aparición de datos faltantes. En los eventos de salud que se encontró que podían ser investigados de manera más detallada, se estimaron razones de prevalencia (RP) crudas, con sus respectivos intervalos de confianza de 95 %, para evaluar su asociación con los grupos de exposición.

Además de estos análisis, y dado que era difícil verificar si se repetían o no los casos de familiares fallecidos, la aproximación a la mortalidad se hizo mediante listados libres.

Este método es de uso principalmente antropológico, aunque se fundamenta en el estudio clásico de psicología experimental de Bousfield y Barclay ^15^. En epidemiología tiene diversos usos, pero el que se buscó en este caso fue el de identificar perfiles epidemiológicos de mortalidad, con base en el conocimiento lego ^16^. El instrumento permitió recolectar datos de hasta cinco fallecidos, de tal manera que el orden en que eran enunciados, y las veces en que se repetía entre todos los informantes, permitió generar el indicador de relevancia denominado “*salience*” de Smith ^17^. Esta aproximación no muestra la frecuencia del evento, sino su importancia para la comunidad ^18^.

### Identificación y selección de eventos con potencial para profundizar

Los eventos de salud de origen ambiental no son fáciles de identificar. Por un lado, pueden ser muy específicos de algunos agentes ambientales (unicausales), pero de muy baja ocurrencia; la otra opción es que sean más frecuentes, pero con varios factores asociados (multifactoriales) ^19^. En el caso de los hidrocarburos presentes en el petróleo crudo, no existen eventos del primer tipo, por lo que su identificación se fundamenta en establecer su mayor ocurrencia entre quienes tienen una exposición mayor; en este caso, esto indica que debe ser mayor entre quienes viven en los resguardos en territorios ancestrales.

De acuerdo con el objetivo del estudio, este se enfocó solo en estos casos, pues cuando había diferencias que mostraban mayor frecuencia en zonas sin exposición, estas se descartaron para su análisis posterior.

Además, teniendo en cuenta las limitaciones temporales, metodológicas y presupuestales, los eventos de interés para un estudio más detallado debían ser detectables mediante una anamnesis sencilla y un examen físico practicado por un médico, sin el apoyo de exámenes paraclínicos.

**Figura 2:**
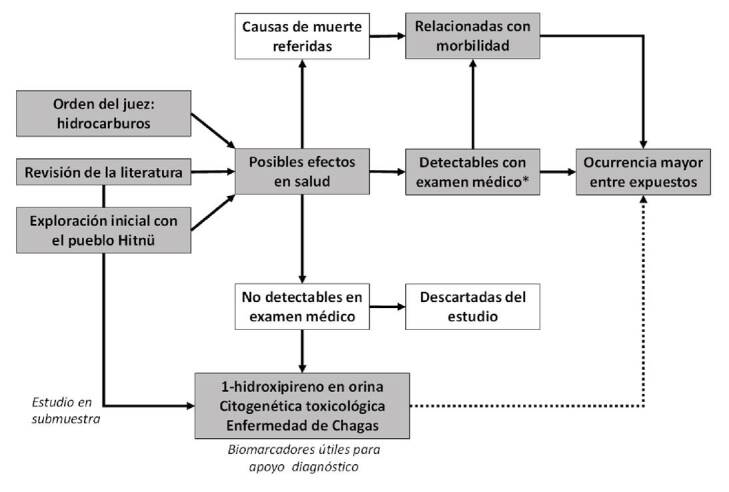
Proceso observado para la identificación de eventos en salud potencialmente asociados con la exposición a hidrocarburos y con posibilidades de ser investigados

El proceso seguido para identificar y seleccionar los eventos potencialmente asociados con la exposición se muestra en la figura 2. Se escogió explorar la ocurrencia de cáncer, efectos reproductivos y efectos neurotóxicos, principalmente. Con los signos y síntomas reportados, los médicos pudieron llegar a una impresión diagnóstica que fue complementada con análisis de los datos clínicos para obtener los diagnósticos sindromáticos ^20^, que podían servir para hacer el diagnóstico diferencial.

Nótese que, además de lo anterior, se tuvieron en cuenta las posibles causas de muerte, y las que pudieran mejorar el diagnóstico médico mediante el uso de tres biomarcadores definidos *a priori*.

El 1-hidroxipireno fue seleccionado por ser el mejor biomarcador de exposición a hidrocarburos del petróleo; la citogenética toxicológica es un biomarcador de sensibilidad importante cuando hay exposición a hidrocarburos, dado su potencial cancerígeno ^21^. Por último, la enfermedad de Chagas también fue seleccionada, debido a que la región donde vive la población hitnü se considera endémica ^22^, lo cual podría tener un efecto sinérgico o antagónico con la exposición a hidrocarburos, de manera similar a lo observado con la malaria y la exposición a mercurio ^23^. Con este procedimiento se buscó evitar lo que, en jerga epidemiológica, se conoce como “expedición de pesca” (*fishing expedition*), que puede llevar a falsos positivos ^24^ y disminuir la eficiencia del estudio.

### Otras consideraciones interculturales

Siguiendo los lineamientos del CONSIDER *statement* para estudios relacionados con personas indígenas ^25^, se intentó tener en todo momento el máximo respeto por la cultura del pueblo hitnü, apoyándose con traductores en los casos en que fue necesario. En relación con la gobernanza, primero se siguieron los pasos sugeridos por las comunidades indígenas de la región para contactarlos, y se tuvo especial consideración en identificar las autoridades tradicionales para que asumieran su rol protagónico.

En las reuniones comunitarias del estudio, se explicaron detalladamente todas las actividades por realizar, haciendo énfasis en los beneficios y los posibles riesgos de participar; fue claro en todo momento que el estudio fue priorizado por ellos mismos, dadas las carencias señaladas por el juez que ordenó la realización del estudio. Las actividades del estudio se explicaron oralmente en reuniones comunitarias y se solicitó consentimiento individual a los participantes para las diversas actividades. Los hallazgos obtenidos en el estudio fueron informados a la población hitnü, teniendo en cuenta las diferencias culturales, y se tuvo una reunión especial con las autoridades para explicarles en mayor detalle y, finalmente, una reunión conjunta con las autoridades, los encargados de su atención en salud y las autoridades de otros pueblos indígenas de la región.

Dado que el trabajo de campo se llevó a cabo en medio de la pandemia de COVID-19, cuando aún no se había iniciado la vacunación, y es bien conocido el potencial efecto adverso que los microorganismos pueden tener en la salud de los indígenas, el equipo investigador tuvo especial cuidado en desarrollar las actividades siguiendo protocolos de bioseguridad. El seguimiento durante varias semanas después del encuentro entre los individuos del pueblo hitnü y los investigadores en campo, permitió verificar que no hubo casos incidentes en ninguno de los grupos.

### Consideraciones éticas

Este estudio fue aprobado por el Comité de Ética en Investigación Científica de la Universidad Industrial de Santander (Acta del 5 de febrero de 2021), después de que los investigadores lograron obtener aprobación oral para la realización del estudio por parte de representantes del pueblo hitnü, en una reunión presencial a la que asistieron dos de los investigadores.

## Resultados

### Caracterización de los asentamientos

Antes de presentar las características de los grupos de estudio, es importante señalar los asentamientos y el número de hogares (nh) que los conforman. En el grupo localizado en la cabecera de Arauca, se incluyeron: Bello Horizonte (nh=6), Matevenado (nh=5), Villa Esperanza (nh=1), Caño Jesús (nh=5), Mategallina (nh=3) y El Paraíso (nh=14). Por su parte, los resguardos ancestrales incluyeron los asentamientos de Las Vegas (nh=21), Gavanes (nh=5), Providencia (nh=3), Trapiche (nh=8), Monogarra (nh=13), Caño Azul (nh=4), Yagual (nh=7), Rodesia (nh=8) y La Ilusión (nh=14). El resguardo de Aspejená se encuentra en un grupo independiente de análisis con 30 hogares.

En el cuadro 1 se presentan las características principales de las viviendas. Como se puede apreciar, se observan cambios asociados con el proceso de sedentarización de un grupo nómada; se pasa de viviendas con material vegetal y sin paredes, al uso de material artificial ^26^. Es evidente que no abunda la infraestructura para tener agua potable, ni para defecar u orinar. La cocina, pese a los cambios, la mayoría de las veces es al aire libre, aunque hay una tendencia a tener un lugar cerrado diferente al de dormir, de acuerdo con la sedentarización; el combustible sigue siendo la biomasa, y cocinar es una actividad de las mujeres de manera predominante.

**Cuadro 1:** Principales características de las viviendas, según regiones con exposición potencial a hidrocarburos del petróleo proveniente del pozo Caricare, establecidas para el estudio (n=147)

Variable		No expuestos		Expuestos	
		Cabecera de Arauca (n=34)	Resguardo Aspejená (n=30)	Resguardo San José del Lipa y La Vorágine (n=83)	p
Paredes (%)					
	Ladrillo	0	0	6,25	
	Guaduas, palos, madera o palma	3,70	75,86	33,75	
	Caucho, plásticos o cartón	81,48	20,69	10	
	Cinc o metal	3,70	0	0	
	Sin paredes	11,11	3,45	50	<0,001*
Piso (%)					
	Tierra	90,91	100	91,25	
	Madera	0	0	6,25	
	Cemento	9,09	0	2,50	0,096*
Techo (%)					
	Plástico	64,71	3,33	1,20	
	Madera o palma	5,88	23,33	89,16	
	Cinc o metal	29,41	73,33	9,64	<0,001
Acueducto^a^ (%)		2,94	0	0	0,435
	Lugar para orinar/defecar (%)				
	Monte	85,29	100	100	
	Hueco o letrina	5,88	0	0	
Inodoro		8,82	0	0	0,002*
Lugar de cocina (%)					
	Aire libre	82,35	66,67	57,83	
	Cerrado; aparte para cocinar	14,71	33,33	26,51	
	Cerrado; donde duermen.	2,94	0	10,84	
	No cocinan en su vivienda.	0	0	4,82	0,073*
Combustible para cocinar (%)					
	Madera	94,12	100	98,80	
	Gas	5,88	0	1,20	0,240*
Mujeres cocinan (%)		88,24	90	95,18	0,321*
Menores de edad cocinan (%)		14,71	6,67	13,25	0,620*
Hombres cocinan (%)		8,82	33,33	27,71	0,035*
Agua para consumo (%)					
	Acueducto	20,59	0	1,20	<0.001*
	Bomba manual (“puntillo”)	73,53	100	44,58	<0.001*
	Jagüey (zanja con agua)	2,94	0	36,14	<0,001*
	Río o caño	0	0	24,10	<0,001*
Tratamiento del agua (%)		5,88	23,33	22,89	0,062*
Falta agua para consumo (%)		70,59	73,33	74,70	0,901
Agua con mal sabor (%)		32,35	40	69,88	<0,001*
Agua con mal olor (%)		17,65	26,67	66,27	<0,001
Agua de color oscuro (%)		14,71	30	61,45	<0,001
Tienen cultivos (%)		29,41	83,33	86,75	<0,001
Cazan y pescan (%)		23,53	76,67	85,54	<0,001
Crían aves (%)		26,47	83,33	68,67	<0,001
Crían ganado vacuno^b^ (%)		0	3,33	27,71	<0,001*

^a^ Esta variable tiene una distribución similar a la del servicio eléctrico y el alcantarillado (no se presentan los datos).

^b^ Esta variable tiene una distribución similar a la de la cría de vacunos (no se presentan los datos).

En relación con el consumo de agua, que puede ser el vehículo en que llegan los hidrocarburos a los indígenas que habitan en los resguardos ancestrales, hay diferencias relevantes entre los tres grupos.

En la cabecera, se tiene mayor variedad de fuentes de agua y han dejado de usar el río. En Aspejená, toda el agua es subterránea, por lo que se usan bombas manuales para su extracción. Por último, en los resguardos ancestrales, se mantiene el río Ele y los caños, junto a las bombas manuales y los jagüeyes, como fuentes de agua para consumo. Resulta llamativo que la mayoría bebe agua sin ningún tipo de tratamiento; en ninguna vivienda se informó que se hierva el agua antes de consumirla. En los asentamientos de los resguardos se observaron cultivos, mas no agricultura en sentido estricto ^27^; son importantes los cultivos de plátano (75,51 %), yuca (74,15 %), maíz (69,39 %) y batata (56,46 %). También, se crían animales, con mayor frecuencia aves como las gallinas.

### Caracterización de los participantes

Con base en el propio censo llevado a cabo durante el trabajo de campo, en este estudio participaron 86,93 % (572/658) de los miembros del pueblo hitnü; la mayoría de los no participantes estaban trabajando en labores agrícolas fuera de los asentamientos, de acuerdo con la información brindada por sus familiares.

En el cuadro 2 se resumen algunas características de los individuos participantes. Como se puede apreciar, es un grupo con proporciones similares entre mujeres y hombres, de edad muy joven en su gran mayoría. El analfabetismo es alto, y el nivel de escolaridad de la población no suele sobrepasar la primaria. Se dedican a diversas actividades, y las que tienen que ver con el sustento alimentario todavía son mayoritarias. Consumen alcohol (principalmente en forma de vinete), tabaco y yopo; este último se mantiene como algo principalmente ritual entre los mayores.

**Cuadro:** 2. Características de los indígenas hitnü participantes en el estudio, según grupos de potencial exposición a los hidrocarburos del petróleo extraído en el pozo Caricare (n=572)^¥^

Variable		n	No expuestos		Expuestos	p
			Cabecera de Arauca (n=34)	Resguardo Aspejená (n=30)	Resguardo San José del Lipa y La Vorágine (n=83)	
Sexo: mujeres (%)		572	73 (53,68)	42 (45,65)	177 (51,45)	0,479
	Edad (años)					
	Mediana	565	14	18	12	0,175
	Percentil 75		27	30	30	
	(mínimo-máximo)		(0,08 - 78)	(1 - 69)	(0,25 - 70)	
	Sin dato*		1	0	10	
Alfabetismo (%)		274	36 (54,55)	38 (65,52)	66 (44,00)	0,017
Educación						
	Sin estudios (%)	409	21 (24,42)	13 (18,31)	84 (33,33)	0,009
	Primaria (%)		46 (53,49)	36 (50,70)	118 (46,83)	
	Secundaria (%)		16 (18,60)	17 (23,94)	29 (11,51)	
	Técnicos/tecnológicos (%)		2 (2,33)	3 (4,23)	3 (1,19)	
	Prescolar, sin estudios (%)		1 (1,16)	2 (2,82)	18 (7,14)	
Ocupación						
	Cazador (%)	245	12 (26,67)	26 (47,27)	87 (60,00)	<0,001
	Pescador (%)	248	12 (27,27)	24 (43,64)	96 (64,43)	<0,001
	Agricultor (%)	247	10 (22,22)	35 (66,04)	99 (66,44)	<0,001
	Artesano (%)	241	10 (20,83)	12 (26,09)	53 (36,05)	0,101
	Cuidado de hijos (%)	247	32 (64,00)	29 (61,70)	96 (64,00)	0,958
	Cría de animales (%)	239	4 (9,30)	36 (70,59)	81 (55,86)	<0,001
Consumos adictivos						
	Alcohol^a^ (%)	562	16 (12,12)	26 (28,26)	136 (40,24)	<0,001
	Tabaco (%)	560	18 (13,74)	11 (11,96)	86 (25,52)	0,002
	Yopo^b^ (%)	560	10 (7,63)	3 (3,26)	62 (18,40)	<0,001
Hallazgos clínicos de desnutrición						
	Cabello opaco (%)	528	42 (35,29)	44 (48,89)	206 (64,58)	<0,001
	Cabello rojizo (%)	533	4 (3,31)	10 (11,11)	79 (24,53)	<0,001
	Emaciación (%)	531	12 (10,00)	8 (8,89)	8 (2,49)	0,002
	Coiloniquia (%)	532	14 (11,57)	8 (8,89)	23 (7,17)	0,328
	Grasa subcutánea ausente (%)	533	1 (0,83)	1 (1,11)	3 (0,93)	1
	Cara edematosa (%)	533	3 (2,48)	3 (3,33)	12 (3,73)	0,811
	Cara enflaquecida (%)	529	2 (1,65)	4 (4,49)	3 (0,94)	0,072
	Edema abdominal (%)	531	11 (9,17)	8 (8,89)	6 (1,87)	0,001
Signos potencialmente neurotóxicos						
	Prueba índice-nariz (%)	498	0 (0,00)	0 (0,00)	14 (4,56)	0,014
	Signo de Romberg (%)	499	0 (0,00)	0 (0,00)	15 (4,89)	0,010
	Nistagmus (%)	529	0 (0,00)	0 (0,00)	3 (0,93)	0,749
	Temblor al movimiento (%)	533	0 (0,00)	0 (0,00)	1 (0,31)	1
	Convulsiones	269	0 (0,00)	6 (18,75)	19 (10,73)	0,001
(%) Salud reproductiva						
	Abortos espontáneos (%)	140	9 (25,71)	3 (12,00)	25 (31,25)	0,162
	Muerte de hijo (%)	136	9 (26,47)	5 (20,00)	26 (33,77)	0,385
	Dificultad para lograr embarazo (%)	108	4 (13,33)	4 (21,05)	17 (28,81)	0,255
Menstruación regular (%)		106	23 (88,46)	23 (100)	55 (96,49)	0,134
Otros hallazgos clínicos						
	Tiroides palpable (%)	517	44 (36,97)	21 (23,86)	95 (30,65)	0,129
	Masa en cuello (%)	519	7 (5,83)	5 (5,68)	70 (22,51)	<0,001
	Masa en abdomen (%)	526	0 (0,00)	2 (2,22)	2 (0,63)	0,171
	Sangrado nasal (%)	527	0 (0,00)	1 (1,14)	0 (0,00)	0,167
	Soplos cardíacos (%)	532	3 (2,48)	3 (3,33)	7 (2,18)	0,822
	Hipoventilación torácica (%)	523	3 (2,56)	3 (3,33)	1 (0,32)	0,038
	Dolor abdominal a la palpación (%)	530	1 (0,84)	12 (13,33)	22 (6,85)	0,001

^¥^ Algunos participantes no brindaron todas las respuestas, por lo que el tamaño de muestra no fue constante entre las variables. a Lo más frecuente es en forma de vinete, bebida alcohólica elaborada a partir de la savia del árbol de palma real (*Scheela roystonea regia*).

^b^ Polvo alucinógeno de color verde-amarillo para uso inhalado; se basa en semillas de *Anadenanthera peregrina*, mezclado con plátano, miel y caracol, entre otros; su principal uso es ritual.

^*^ Algunos indígenas hitnü no conocen su edad o pueden no conocerla con exactitud.

### Perfil de morbilidad

Hubo signos y síntomas de baja ocurrencia que fueron descartados para la descripción y los posteriores análisis (no se muestran por su extensión). A continuación, se presentan algunos hallazgos que sirven para evidenciar el perfil general de morbilidad, y de eventos potencialmente asociados con la exposición a los hidrocarburos.

En relación con los síntomas, los cuales son muy variados, es notorio que la cefalea, la fiebre, las palpitaciones, la hematuria, la disnea, el dolor abdominal y la diarrea, ocurran principalmente en los asentamientos no urbanos. Otras manifestaciones clínicas, como: poliuria, picazón en la nariz, rinorrea, estornudos, faringitis, tos y flemas, son más frecuentes en los resguardos ancestrales.

En relación con los signos identificados por los médicos, fue notorio que la emaciación y la hipoventilación torácica fueron más frecuentes entre quienes viven en zonas urbanas o el resguardo de Aspejená, en comparación con los territorios ancestrales.

El cabello opaco o rojizo, las masas en el cuello, la prueba índice-nariz fallida y el signo de Romberg, se observaron más frecuentemente entre quienes viven en los resguardos ancestrales.

En relación con los eventos reproductivos, es evidente una mayor ocurrencia de abortos, muerte de hijos, y dificultades para lograr el embarazo, entre quienes viven en los resguardos. Sin embargo, las diferencias no fueron estadísticamente significativas, quizá por el tamaño reducido de la muestra con respecto a las mujeres que respondieron estas preguntas.

Al expresar las asociaciones con la relación de proporción (RP) y tomando como referencia el grupo que está en zonas urbanas, se observó que las masas en el cuello detectadas por los médicos se asociaban con el grupo de exposición en los resguardos ancestrales (RP=,86; IC_95%_ 1,77-8,39). Esto concuerda con el autorreporte de “chichones” en el grupo de Aspejená (RP=2,66; IC_95%_ 1,65-4,29); y en el grupo de los resguardos ancestrales (RP=1,92, IC_95%_ 1,28-2,96). No hubo otros eventos asociados con el grupo expuesto, por lo menos en una primera exploración bivariada

### Perfil de mortalidad

Después de la reclasificación, los listados libres permitieron identificar 18 causas de muerte, que incluyeron: causas externas, enfermedades infecciosas y crónicas variadas e, incluso, síndromes de filiación cultural (cuadro 3). Se destaca el homicidio en el primer lugar, lo que denota la importancia que tiene la violencia que ha sufrido el pueblo hitnü; le siguen los tumores, que pueden ser de diferente origen y que podrían asociarse con la contaminación por hidrocarburos o enfermedades infecciosas. En tercer lugar, se encuentra la tuberculosis, seguida por las cardiopatías que pueden ser de origen chagásico, pues se sabe que la enfermedad es endémica en la región y entre los hitnü ^22^. Resulta interesante observar que el alcoholismo y el suicidio ^28^^,^^29^, el hambre ^30^, la mordedura de serpientes y la “brujería”, son frecuentes entre los indígenas colombianos.

**Cuadro 3:** Importancia reportada de las causas de muerte informadas por el jefe del hogar; resultados de listados libres

Causa de muerte	(%)^a^	Posición promedio^b^	Relevancia^¥^	Observaciones
Homicidio	24,4	1,27	0,219	Incluye por guerrilla, paramilitares y ejército, y minas antipersona.
Tumor	13,3	1,17	0,122	Incluye cáncer, “masas” y “chichones”.
Tuberculosis	13,3	1,33	0,115	
Gastrointestinal	8,9	1	0,089	
Cardiopatía	8,9	1	0,089	
No sabe	8,9	1	0,089	
COVID-19	8,9	1,25	0,078	Datos oficiales, no tienen este registro.
Alcoholismo	6,7	1,33	0,056	
Vejez	6,7	1,67	0,052	
Hambre	4,4	1	0,044	
Respiratorio	4,4	2	0,022	
Ofidismo	2,2	1	0,022	Posible mordedura de Bothrops sp.
Suicidio	2,2	1	0,022	
“Brujería”	2,2	1	0,022	
Sangrado interno	2,2	1	0,022	
Muerte materna	2,2	1	0,022	
Ahogamiento	2,2	2	0,015	
Nefropatía	2,2	3	0,007	

^a^ Número de participantes que incluyeron la respuesta.

^b^ Es el valor promedio de la posición en las listas de la respuesta.

^¥^ Valores de indicadores de relevancia (salience) de Smith

## Discusión

Los hallazgos del presente estudio sugieren que, en el perfil de morbimortalidad del pueblo hitnü, tienen preponderancia las enfermedades infecciosas y crónicas, así como las lesiones traumáticas y los problemas nutricionales. Hay una amplia variedad de condiciones de salud, que parecen depender en gran medida del contexto, las cuales sugieren ser consecuencia acentuada de los factores determinantes socioculturales y ambientales. La rápida transición del nomadismo al sedentarismo sin seguridad alimentaria ^31^ y el desplazamiento forzado, parecen ocasionar cambios, y unos de sus efectos más evidentes son la desnutrición, en la región urbana, y el sobrepeso y obesidad entre algunos subgrupos de Aspejená y de los resguardos ancestrales. Esto último parece estar relacionado con la estratificación social interna de cada asentamiento. Es un perfil epidemiológico muy particular, ajeno a lo observado a nivel nacional; se evidencian la pobreza extrema y la falta de acceso a servicios de salud.

Las enfermedades infecciosas están presentes ampliamente, desde las más comunes, como las infecciones respiratorias y gastrointestinales, hasta enfermedades crónicas como la tuberculosis y la enfermedad de Chagas; de esta última se confirmó su alta ocurrencia, similar a la observada 10 años antes ^22^. Su presencia, incluso, es evidencia clara de las condiciones socioambientales precarias y la falta de acceso a servicios de salud de calidad.

Por el interés que motivó este estudio, surge la necesidad de explorar con mayor detalle si las linfoadenopatías observadas con mayor frecuencia en los resguardos relacionados con el río Ele y sus afluentes, indican la presencia de neoplasias. Algunos tumores, según informaron algunos participantes, se han visto en el cuerpo de individuos que han fallecido. Sus allegados no conocen muy bien su causa, y es difícil que lleguen a ser atendidos por médicos; la consecuencia es que no hay registros de mortalidad confiables para los miembros del pueblo hitnü.

Infortunadamente, esto es algo común en este tipo de poblaciones y su consecuencia es el ocultamiento de un hecho sanitario importante. En similar condición se encuentran los hallazgos neurológicos que podrían explicarse por algún agente ambiental presente.

Asimismo, este estudio mostró que el contexto del pueblo hitnü es similar al de otros indígenas de Latinoamérica, donde existen conflictos socioambientales por la extracción petrolera. Quizá los más conocidos son los casos de los mapuches en Argentina; los apurina, deni, juma y paumari en Brasil; los achuar, cofan, quichua, secoya, shuar, siona y waorani en la Amazonía ecuatoriana; los maya en Guatemala y México; los amahuaca, ese’eje, mascho-piro urarina y yoro en Perú; y los warao en Venezuela ^32^.

En Colombia, especial atención tiene el caso de los u’wa, cuyo clímax ocurrió hace casi dos décadas, relacionado con la extracción petrolera en Samoré (Toledo, Norte de Santander). Este grupo indígena se opuso al proyecto debido, entre otros aspectos, a los posibles derrames de hidrocarburos por problemas en los procesos productivos y, sobre todo, por las posibilidades de derrames ocasionados por ataques de los grupos guerrilleros al oleoducto Caño Limón-Coveñas ^33^.

Hallazgos como los descritos anteriormente son similares a los reportados en situaciones parecidas, pero infortunadamente, muy poco estudiados en su mayoría. Esto contrasta con los variados estudios sobre explotación petrolera en la Amazonía ecuatoriana, lo cual hace que su comparación resulte de gran utilidad. El trabajo aquí presentado se realizó en menos de dos años y en tiempos de pandemia, lo que obligó a los investigadores a optimizar los métodos para poder obtener resultados con utilidad para la toma de decisiones.

Esto contrasta con el estudio en Ecuador, el cual, durante varios años utilizó una aproximación participativa siguiendo el modelo de: “epidemiología popular” propuesto por Brown ^34^. Esto se facilitó por el trabajo previo del investigador principal en el territorio, pero el proceso fue complejo y requirió bastante tiempo para tener resultados ^35^. Lo interesante es que los hallazgos son similares a los que aquí se presentaron en relación con la importancia de las neoplasias como posibles efectos de la exposición a los hidrocarburos del petróleo ^36^.

Aunque los resultados fueron objeto de crítica ^37^, sirvieron para una discusión entre epidemiólogos sobre las limitaciones inherentes de la epidemiología ambiental y la ética, al investigar problemas sanitarios en los cuales los afectados son minorías étnicas, con condiciones como dificultad para el acceso por la geografía, y la precariedad de los servicios de salud y educación ^38^.

Por o anterior, los resultados quí presentados corresponden a una aproximación general a la problemática sanitaria del pueblo hitnü que, por el propio diseño del estudio, tiene limitaciones. No obstante, el estudio favoreció la observación de efectos crónicos no letales o asociados con el tiempo de sequía en la región. De esta manera, se excluyeron eventos como los asociados con insectos transmisores de infecciones, infecciones respiratorias y mordeduras de serpientes, que son de alta ocurrencia durante los periodos lluviosos.

Por el diseño, participaron todos los indígenas hitnü que estaban en el asentamiento en el momento del estudio, razón por la cual los resultados son representativos, pese a no tener un muestreo específico para tal fin.

Los resultados no indican que solo las linfoadenopatías requieren ser estudiadas con mayor profundidad para verificar su posible asociación con la exposición a hidrocarburos del petróleo, sino que puede haber más posibles efectos adversos, tal vez de menor ocurrencia o que requieren otras formas de diagnóstico, que se escapan a las limitaciones de un estudio sin apoyos diagnósticos ni consulta con especialistas.

En conclusión, se confirma la necesidad de explorar con más detalle la posible ocurrencia aumentada de linfoadenopatías, asociada con la exposición a los hidrocarburos presentes en el petróleo crudo, identificada mediante RP cruda. Los métodos y resultados descritos en este trabajo sirven de fundamento para un estudio epidemiológico específico sobre ellas, así como la profundización del tema con análisis de citogenética toxicológica y mediciones de exposición a hidrocarburos del petróleo, mediante la detección de 1-hidroxipireno en orina y, así, determinar si las linfoadenopatías pueden ser de origen neoplásico (leucemias, linfomas o metástasis). En esos estudios ya se podrán controlar los posibles factores de confusión en la asociación.

Las limitaciones inherentes a un estudio observacional exploratorio como éste ya son conocidas, así como la necesidad de tomar acciones ante situaciones sanitarias precarias como las de los hitnü. Debe mencionarse, por tanto, que los hallazgos presentados no indican causalidad, pero sí son sugestivos de una posible asociación que debería ser mejor estudiada; además, es evidente que este tipo de poblaciones se encuentra afectada por factores sociales y culturales muy específicos que pueden actuar conjuntamente, y que entender sus interacciones requiere abordajes que van más allá de la epidemiología y las actividades convencionales de salud pública ^39^.

Será importante explorar con mayor detalle los efectos reproductivos adversos más sutiles, como la disminución de la fecundidad y los efectos neurotóxicos que, por su baja ocurrencia o dificultades culturales de medición, generan un reto mayúsculo. De igual manera, es evidente que las condiciones de nutrición y seguridad alimentaria son precarias en una proporción importante de los hitnü, así como la ocurrencia de enfermedades infecciosas como la tuberculosis y la enfermedad de Chagas. Sin embargo, el estudio de la potencial asociación de estas enfermedades con la exposición a hidrocarburos no implica que se detengan acciones e intervenciones urgentes que tengan como objetivo mejorar el agua para consumo, las condiciones de salud, nutrición y seguridad, desde un enfoque intercultural que respete la autonomía del pueblo hitnü.

Dados los resultados, la búsqueda activa de casos sospechosos de cáncer, enfermedad de Chagas, tuberculosis y desnutrición, fue solicitada por el equipo investigador a la IPS-I encargada de la atención de esta población, a la EPS, a las autoridades locales y al Ministerio de Salud y Protección Social. Se ha transmitido de manera clara, la falta de atención en salud que está poniendo en peligro de extinción a la cultura hitnü, y que, por ser un pueblo de especial protección constitucional, las autoridades deberían vigilar estrechamente dichas actividades y no simplemente delegar responsabilidades a los prestadores de salud y aseguradora.

Los hallazgos aquí descritos corresponden a un claro caso de injusticia ambiental ^40^, en el que uno de los grupos más desfavorecidos de Colombia puede estar siendo afectado por una suma de factores determinantes socioculturales y ambientales. Estos hallazgos serán presentados por el Ministerio de Salud y Protección Social ante las autoridades judiciales, para que se tomen acciones que busquen la protección del pueblo hitnü y sirvan de modelo para estudios interculturales de salud ambiental que se realicen en el país.
